# Knowledge attitudes and practices regarding MRI safety among healthcare providers and patients/family members in China

**DOI:** 10.1038/s41598-026-44648-5

**Published:** 2026-03-23

**Authors:** Mengdi Zhang, Gaofeng Lu, Dongzhi Zhai, Yanna Guo, Zhenzhen Li, Yitong Xing

**Affiliations:** 1 Department of Medical Imaging, The Second Affiliated Hospital of Zhengzhou University, Zhengzhou, 450014 China; 2 Department of Gastroenterology, The Second Affiliated Hospital of Zhengzhou University, Zhengzhou, 450014 China

**Keywords:** Magnetic resonance imaging, Safety, Knowledge, Attitudes, Practice, Healthcare providers, Patients and accompanying family members, Cross-sectional study, Diseases, Health care, Medical research

## Abstract

Evidence regarding knowledge, attitudes, and practices (KAP) related to MRI safety among healthcare providers and the public in China remains limited. A web-based cross-sectional study. HCPs (including clinicians, nurses, MRI technologists, and medical students) completed the survey based on their experience in MRI-related environments, whereas PFs completed the survey as examinees/patients and accompanying family members/caregivers. Data were collected between March 1, 2024, and May 1, 2025. A total of 924 questionnaires were collected, of which 46 were excluded due to lack of informed consent, insufficient response time, age ineligibility, or data outliers, resulting in 878 valid questionnaires. HCPs demonstrated higher knowledge, attitude, and practice scores than PFs. Structural equation modeling showed that knowledge significantly influenced attitudes in both groups, while attitudes exerted a strong direct effect on practice. Both groups exhibited limited knowledge and moderate attitudes toward MRI safety. These findings suggest potential implications for future educational strategies aimed at improving MRI safety awareness and compliance.

**Supplementary Information:**

The online version contains supplementary material available at 10.1038/s41598-026-44648-5.

## Introduction

### MRI Safety and clinical risk background

Magnetic Resonance Imaging (MRI) serves as a crucial diagnostic modality in contemporary healthcare. Compared with conventional imaging techniques such as computed tomography (CT) and X-ray radiography, MRI provides superior soft tissue contrast, allowing clearer visualization of organs, muscles, nerves, and soft tissue structures without the use of ionizing radiation^1^. This advantage makes MRI particularly valuable for neurological, musculoskeletal, and abdominal imaging, while also reducing radiation-related risks associated with repeated examinations. While MRI is predominantly regarded as a safe imaging technique, it incorporates three distinct electromagnetic fields: the static magnetic field (B0), the radiofrequency field (B1), and time-varying gradient magnetic fields. Each of these fields poses unique safety risks that require meticulous management^2^. As MRI technology has continued to advance, new safety challenges have also emerged. Modern MRI systems provide higher image resolution and faster scanning performance; however, these improvements are accompanied by stronger magnetic environments and more complex operational procedures. As a result, the risk of safety incidents, such as projectile accidents caused by ferromagnetic objects, thermal injuries, and device-related complications, has increased, potentially leading to patient harm and, in rare cases, serious adverse outcomes^3^. Beyond technological factors, human-related elements such as awareness, perceptions, and behavioral compliance play a critical role in preventing MRI-related adverse events, highlighting the need to evaluate safety performance from a behavioral perspective.

### Conceptual framework: KAP and MRI safety screening

The Knowledge, Attitude, and Practices (KAP) survey methodology provides a systematic framework for understanding the interplay between knowledge, attitudes, and behaviors within healthcare environments. In previous studies, this approach has been widely applied to evaluate how individuals’ awareness of health-related information influences their perceptions and subsequently shapes real-world behavioral compliance, thereby helping to identify key targets for educational and behavioral interventions^4,5^. This framework is particularly relevant to MRI safety, where the secure operation of MRI necessitates a multi-faceted screening process encompassing pre-procedure questionnaires, day-of-examination interviews, and final safety checks^2^. The effectiveness of these procedures has been shown to depend strongly on patients’ understanding, accurate self-reporting of medical history and implants, active cooperation during safety screening, as well as the assistance of accompanying family members, in addition to institutional safety management and staff supervision^2,6^. With estimates suggesting that 10–20% of MRI patients possess implanted medical devices^7^, along with emerging risks such as metallic microfibers in clothing and metal components in face masks, the intricacy of safety screening continues to escalate. In routine clinical practice, patients undergoing MRI examinations are required to complete standardized safety screening forms, participate in face-to-face safety interviews, and comply with pre-scan preparation procedures, including the removal of metallic objects and verification of implanted devices. Accompanying family members or caregivers may also be involved in assisting patients during registration, preparation, and safety screening processes. Therefore, patient- and caregiver-related MRI safety behaviors are shaped not only by individual knowledge but also by institutional procedures and communication with healthcare staff. Previous studies have reported that patients frequently experience anxiety, misunderstanding of MRI-related risks, and insufficient awareness of safety precautions, which may negatively affect cooperation during screening and scanning procedures^8,9^. Concerns related to noise, confined spaces, contrast agents, and implanted devices are commonly reported barriers to effective patient compliance^10,11^. These findings indicate that patient-centered safety education and behavioral guidance play a critical role in reducing preventable MRI-related adverse events and improving examination safety.

### MRI safety challenges in China

In the context of China, the availability and use of MRI scanners have increased markedly over recent decades. The number of MRI machines in China has risen substantially, with reported configurations exceeding 6,000 units by 2017, reflecting sustained expansion of high-technology imaging resources nationwide^12^. Studies examining MRI service use also indicate a sharply increasing trend in MRI utilization in Chinese hospitals following health system reforms^13^. However, this rapid expansion is accompanied by variability in professional training capacity and uneven patient health literacy, which may challenge the consistency of MRI safety screening and communication in routine practice. International research indicates that approximately 60% of MRI accidents stem from oversights in screening procedures^6^, whereas the standardization of screening protocols in grassroots Chinese hospitals falls below 40% ^14^. Comparative analyses reveal considerable knowledge gaps, with 38–52% of healthcare professionals globally lacking a comprehensive understanding of MRI safety principles^15,16^. Investigations conducted in singular centers in China have underscored systemic issues, including 62% of safety screening documentation being incomplete and 28% of ferromagnetic objects being misidentified^17^. Frameworks pertaining to patient safety culture suggest that effective interventions must address deficits in knowledge, attitudes, and organizational factors^18^. Despite these challenges, research exploring KAP related to MRI safety in China remains sparse.

### Study rationale and objectives

Accordingly, this study primarily assessed MRI safety–related KAP from the perspective of individuals undergoing MRI examinations and accompanying caregivers (PFs), while healthcare providers (HCPs) were included as a comparison group to examine differences associated with occupational background in MRI-related safety awareness and behaviors.

This study seeks to bridge these gaps by conducting a comprehensive KAP assessment focusing on MRI safety awareness and behaviors among patients and accompanying family members in China. Based on the KAP theoretical framework, this study aimed to address the following research hypotheses: (1) knowledge is positively associated with attitude toward MRI safety; (2) attitude is positively associated with MRI safety–related practice; and (3) knowledge has both direct and indirect effects on practice through attitude in both HCP and PF groups.

## Methods

### Study design and participants

This cross-sectional study was conducted at the Second Affiliated Hospital of Zhengzhou University between March 1, 2024 and May 1.2025. Participants were recruited through convenience sampling and were categorized into two groups: healthcare providers (HCPs; including clinicians, nurses, MRI technologists, and medical students) and patients/families (PFs; examinees/patients and accompanying family members/caregivers). The study protocol received approval from the Ethics Committee of the Second Affiliated Hospital of Zhengzhou University (Ethics Approval Number: NO.KY2024145), and informed consent was obtained from all participants prior to the commencement of data collection.

### Procedures

A self-administered questionnaire was developed in accordance with established MRI safety guidelines and pertinent literature^14,19–21^. To ensure content validity, a panel comprising three radiologists and three MRI technologists, each possessing a minimum of five years of professional experience, reviewed the questionnaire, resulting in minor revisions informed by their feedback. A pilot test involving 30 participants was conducted to assess the clarity and reliability of the instrument. The Cronbach’s alpha values for the knowledge, attitude, and practice sections were recorded at 0.814, 0.845, and 0.901, respectively, indicating a high level of internal consistency. In addition to internal consistency assessment, confirmatory factor analysis (CFA) was performed separately for healthcare providers (HCPs) and patients/family members (PFs) to evaluate the adequacy of the measurement model prior to structural equation modeling. For HCPs, the CFA model demonstrated acceptable fit, with RMSEA = 0.055, SRMR = 0.057, TLI = 0.892, and CFI = 0.904 (**Supplementary Fig. 1**). For PFs, the measurement model also showed adequate fit, with RMSEA = 0.064, SRMR = 0.061, TLI = 0.881, and CFI = 0.894 (**Supplementary Fig. 2**). These results supported the suitability of the measurement structure for subsequent structural model estimation. The questionnaire items were designed to reflect common MRI examination scenarios, including pre-examination safety screening, on-site safety reminders, patient cooperation behaviors, and interactions with MRI staff during routine clinical workflows. Healthcare providers were instructed to complete the questionnaire based on their experience in MRI-related environments (including workplace exposure and MRI safety screening procedures), whereas patients and accompanying family members were asked to respond according to their personal experience during MRI examinations and safety screening procedures. The questionnaire did not assess clinical decision-making or patient management behaviors.

The finalized version of the questionnaire, administered in Chinese, encompassed four dimensions totaling 47 items. The basic information section comprised 16 items, while the knowledge, attitude, and practice dimensions contained 14, 12, and 5 items, respectively (Questionnaire). In the PFs group, respondents were categorized as either patients undergoing MRI examinations or accompanying family members/caregivers, and both groups completed the same questionnaire based on their participation in MRI-related safety screening and examination processes. All data were collected and analyzed at the individual level; no family-level aggregated measures were constructed. A structured scoring system was employed for each dimension. The knowledge section utilized a three-tiered scoring approach: correct responses were awarded 2 points, uncertain (“not sure”) responses received 1 point, and incorrect responses were assigned 0 points, resulting in a total possible score ranging from 0 to 28. This scoring strategy was designed to reflect graded levels of cognitive familiarity, in which uncertainty represents partial awareness that is conceptually distinct from both accurate knowledge and complete lack of knowledge, thereby allowing a more nuanced assessment of respondents’ knowledge status. The attitude and practice dimensions were evaluated using a five-point Likert scale, with response options varying from “Strongly Agree” (5 points) to “Strongly Disagree” (1 point). The total score for the attitude dimension ranged from 12 to 60, while the practice dimension scores ranged from 5 to 25. To enhance data interpretation, knowledge levels were classified as insufficient (0–14 points), moderate (15–21 points), and adequate (22–28 points). Attitudes were categorized as negative (12–30 points), neutral (31–45 points), and positive (46–60 points). Similarly, practice behaviors were classified as negative (5–13 points), moderate (14–19 points), and positive (20–25 points)^22^. For regression analysis, participants with practice scores ≤ 19 were categorized as the “low practice” group, whereas those with scores ≥ 20 were categorized as the reference group.

### Questionnaire distribution and quality control

A convenience sampling strategy was employed to recruit HCPs and PFs. Data collection was conducted through an online questionnaire developed within the WeChat-based Wenjuanxing mini-program. Participants accessed the survey by scanning a QR code disseminated via WeChat, facilitating the electronic completion of the questionnaire. To enhance data quality and mitigate the risk of duplicate submissions, each IP address was restricted to a single response, and all questions were designated as mandatory. The research team systematically reviewed the submitted questionnaires for completeness, internal consistency, and logical coherence. Invalid responses were excluded based on the following criteria: (1) a response time of less than 90 s, (2) logically inconsistent answers, and (3) identical responses across all items within any KAP dimension. The 90-second threshold was determined according to the questionnaire length (47 items) and the median completion time observed during pilot testing, and was used to exclude responses that were unlikely to reflect careful reading and thoughtful answering. Similar response-time–based controls (e.g., identifying “speeding” on web surveys via minimal completion time thresholds) have been described in the online survey methodology literature to reduce inattentive responding and enhance data quality^23^.

### Sample size calculation

The sample size was estimated prior to data collection to ensure sufficient statistical power for reliable analysis. Given that the questionnaire contained 47 substantive items across the knowledge, attitude, and practice dimensions, the commonly used guideline of 5 to 10 participants per item was applied^24^. This approach suggested a total required sample size of 235 to 470 participants to achieve stable estimates and support robust statistical analysis. To account for potential non-responses and incomplete submissions, an additional 10% buffer was incorporated into the initial target, leading to a planned recruitment of approximately 260 ~ 523 participants. Ultimately, the final number of valid respondents exceeded the initially planned sample size, ensuring adequate statistical power for subgroup analyses and structural equation modeling.

### Statistical analysis

Statistical analysis was conducted using Statistical Package for the Social Sciences (SPSS) version 27.0 (IBM Corp., Armonk, NY, USA) and Analysis of Moment Structures (AMOS) version 26.0 (IBM Corp., Armonk, NY, USA). Continuous variables were assessed for normality and described as means with standard deviations (SD) or medians with interquartile ranges (IQR), depending on the distribution. Categorical variables were presented as frequencies and percentages (n, %). Continuous variables were assessed for normality prior to analysis. Because the total KAP scores did not meet normality assumptions across subgroups, non-parametric tests were applied. Between-group comparisons were conducted using the Wilcoxon-Mann-Whitney test for two-group comparisons and the Kruskal–Wallis test for multiple-group comparisons. Correlation analyses employed Pearson or Spearman correlation coefficients based on data distribution. Univariate and multivariate regression analyses were conducted to explore associations between demographic variables and proactive practices. The proactive practice was defined as achieving a score equal to or greater than 80% of the maximum possible practice score. A structural equation modeling (SEM) analysis was performed to observe the correlations among KAP. All variables derived from five-point Likert-scale items were treated as continuous variables in the SEM analysis. This approach was adopted because Likert scales with five or more response categories and approximately symmetric distributions can be reasonably modeled as continuous variables in structural equation modeling. Model estimation was conducted using the robust maximum likelihood (MLR) estimator to account for potential mild deviations from multivariate normality. The MLR estimator provides robust standard errors and adjusted chi-square statistics, thereby improving the reliability of parameter estimation under non-ideal distributional conditions. Specifically, the proposed SEM structure consisted of three observed variables (knowledge, attitude, and practice), with directional paths specified from knowledge to attitude, from attitude to practice, and from knowledge directly to practice. All statistical analyses were performed using a two-sided test. Two-sided *P* < 0.05 were considered statistically significant.

## Results

### Sample description and questionnaire quality

A total of 924 questionnaires were initially collected for this study. After applying the exclusion criteria, 46 cases were removed from the dataset: 9 lacked informed consent, 34 had a response time of less than 90 s, 1 was identified as an age outlier, and 2 respondents were under 18. This left a final valid dataset of 878 cases, resulting in an effective response rate of 95.02%. The dataset included 525 responses from healthcare professionals and medical students, along with 353 responses from examinees/patients and accompanying family members or caregivers. The questionnaire exhibited acceptable internal consistency within each subgroup (HCPs and PFs). Among HCPs, the overall Cronbach’s α coefficient was 0.8279, with subscale values of 0.6750 for knowledge, 0.6826 for attitude, and 0.8755 for practice. The KMO value for this group was 0.8784. In the subgroup of examinees and their accompanying family members, the questionnaire showed an overall Cronbach’s α of 0.8896, with values of 0.8320 for knowledge, 0.8019 for attitude, and 0.8787 for practice. The KMO value for this subgroup was 0.8858.

### Sociodemographic characteristics by subgroup

#### Healthcare providers (HCPs)

Within the group of 525 healthcare professionals and medical students, 236 were affiliated with the radiology/imaging department, 303 had no formal professional title, and 292 had less than one year of work experience. Among the healthcare providers and medical students (*N* = 525), female respondents made up 63.6% (334), and 57.1% (300) were 25 years old or younger. The majority held bachelor’s degrees (83.6%, 439) and were employed in provincial-level hospitals (57.0%, 299). Notably, radiology staff demonstrated significantly higher knowledge and practice scores. Age significantly affected attitudes; professionals aged 26–45 achieved higher scores. Additionally, marital status was linked to knowledge (*P* = 0.006) and attitude (*P* < 0.001), with married individuals scoring higher. Experience with MRI procedures significantly enhanced practice scores (Table 1).

**Table 1:** Baseline characteristics for healthcare providers (*n* = 525).

	*N*(%)	Knowledge	*P*	Attitude	*P*	Practice	*P*
		Mean (SD)		Mean (SD)		Mean (SD)	
**Total score**	525(100.0)	10.90 (2.00)		37.29 (4.07)		22.34 (2.95)	
**Gender**			0.818		0.647		0.575
Male	191(36.4)	10.74 (2.29)		37.20 (3.94)		22.15 (3.14)	
Female	334(63.6)	10.99 (1.82)		37.34 (4.14)		22.45 (2.84)	
**Age**			0.007		< 0.001		0.117
25 or below	300(57.1)	10.72 (2.06)		36.56 (3.75)		22.15 (2.91)	
26 ~ 45	199(37.9)	11.15 (1.93)		38.28 (4.16)		22.60 (3.03)	
More than 45	26(5.0)	11.04 (1.73)		38.08 (5.13)		22.58 (2.73)	
**Place of residence**			0.006		< 0.001		0.018
Rural	135(25.7)	10.77 (1.80)		36.07 (3.55)		22.09 (2.81)	
Township	74(14.1)	10.62 (1.82)		37.01 (3.48)		21.89 (2.79)	
Urban	316(60.2)	11.02 (2.12)		37.87 (4.28)		22.55 (3.04)	
**Educational level**			0.468		0.262		0.434
Associate degree/bachelor’s degree	439(83.6)	10.91 (1.91)		37.23 (4.02)		22.40 (2.92)	
Master’s degree or above	86(16.4)	10.84 (2.41)		37.55 (4.30)		22.06 (3.12)	
**Monthly income**			0.080		< 0.001		0.129
< 2000	270(51.4)	10.79 (1.93)		36.56 (3.67)		22.15 (2.89)	
2000–5000	81(15.4)	11.21 (1.88)		38.09 (3.80)		22.69 (2.63)	
5000–10,000	117(22.3)	10.92 (2.29)		37.75 (4.95)		22.25 (3.36)	
> 10,000	57(10.9)	10.93 (1.91)		38.63 (3.58)		22.93 (2.75)	
**Marital status**			0.006		< 0.001		0.255
Unmarried/other	348(66.3)	10.78 (2.01)		36.79 (3.76)		22.26 (2.89)	
Married	177(33.7)	11.14 (1.98)		38.25 (4.46)		22.50 (3.08)	
**Department**			< 0.001		< 0.001		< 0.001
Radiology/Imaging Department	236(45.0)	11.45 (1.64)		38.18 (3.92)		23.04 (2.44)	
Internal medicine	60(11.4)	10.35 (2.82)		36.62 (4.27)		21.63 (3.42)	
Surgery	28(5.3)	10.86 (1.30)		37.25 (2.95)		22.46 (2.97)	
Other department	201(38.3)	10.42 (2.02)		36.44 (4.12)		21.71 (3.17)	
**Professional title level**			0.002		< 0.001		0.024
None	303(57.7)	10.67 (2.14)		36.47 (3.88)		22.09 (3.05)	
Junior	79(15.0)	11.30 (1.86)		38.46 (4.40)		23.04 (2.79)	
Intermediate	108(20.6)	11.12 (1.80)		38.33 (4.08)		22.58 (2.72)	
Associate senior or above	35(6.7)	11.29 (1.38)		38.49 (3.26)		22.20 (2.90)	
**Years of work experience**			0.002		< 0.001		0.128
<1 year	292(55.6)	10.76 (1.93)		36.54 (3.85)		22.11 (3.05)	
1–5 years	63(12.0)	11.19 (2.71)		38.32 (4.20)		22.46 (3.09)	
6–10 years	58(11.0)	10.72 (2.43)		37.29 (4.75)		22.48 (2.58)	
> 10 years	112(21.3)	11.18 (1.37)		38.63 (3.73)		22.81 (2.76)	
**Level of the hospital**			0.044		0.002		0.449
Provincial/ministerial level	299(57.0)	10.87 (1.98)		37.33 (3.83)		22.29 (2.84)	
Municipal level	129(24.6)	10.80 (1.88)		36.67 (4.28)		22.31 (3.16)	
County level	72(13.7)	11.35 (1.91)		38.57 (4.35)		22.72 (3.01)	
Township level or below	25(4.8)	10.52 (2.93)		36.20 (4.09)		22.04 (3.06)	
**Experience of MRI**			0.045		< 0.001		0.001
No	270(51.4)	10.75 (2.10)		36.65 (3.90)		21.96 (3.10)	
Yes	255(48.6)	11.05 (1.88)		37.96 (4.14)		22.75 (2.73)	
**Metallic implants in the body**			0.001		0.314		0.789
No	502(95.6)	10.98 (1.88)		37.33 (4.08)		22.35 (2.93)	
Yes	23(4.4)	9.13 (3.38)		36.26 (3.78)		22.22 (3.38)	
**Understand the material of the metallic implant in the body**			0.005		0.519		0.038
No	9(1.7)	8.67 (4.33)		35.44 (4.56)		20.22 (3.73)	
Yes	14(2.7)	9.43 (2.74)		36.79 (3.26)		23.50 (2.50)	
**Condition**			0.002		0.063		0.521
One of the above	45(8.6)	10.07 (2.49)		35.71 (5.21)		21.98 (3.20)	
None of the above	480(91.4)	10.98 (1.94)		37.43 (3.92)		22.38 (2.93)	
**History of drug allergies**			0.128		0.125		0.287
No	497(94.7)	10.94 (1.95)		37.37 (3.97)		22.37 (2.94)	
Yes	28(5.3)	10.18 (2.70)		35.71 (5.32)		21.79 (3.13)	

### Patients and accompanying family members (PFs)

The PFs group (*N* = 353), including patients and accompanying family members/caregivers, exhibited a balanced gender distribution, with 52.1% identified as male (184 participants). Among this group, 47.6% (168 participants) were aged 26 to 45 years. Educational attainment significantly influenced knowledge, as individuals with bachelor’s degrees scored higher. Income levels correlated with attitudes (*P* = 0.006); participants earning over 10,000 CNY per month reported elevated scores. Married participants showed enhanced knowledge and practice. Previous experience with MRI was associated with improved knowledge and attitudes, while a history of drug allergies adversely affected practice scores (Table 2).

**Table 2:** Baseline characteristics for patients and accompanying family members (*n* = 353).

	*N*(%)	Knowledge	*P*	Attitude	*P*	Practice	*P*
		Mean (SD)		Mean (SD)		Mean (SD)	
**Total score**	353(100.0)	7.34 (3.45)		33.65 (4.39)		19.94 (4.02)	
**Gender**			0.145		0.114		0.160
Male	184(52.1)	7.49 (3.62)		33.95 (4.54)		20.17 (4.31)	
Female	169(47.9)	7.18 (3.27)		33.33 (4.22)		19.69 (3.66)	
**Age**			0.031		0.001		< 0.001
25 or below	57(16.1)	7.04 (3.63)		32.39 (4.10)		19.07 (3.10)	
26 ~ 45	168(47.6)	7.77 (3.45)		34.56 (4.34)		20.97 (3.76)	
More than 45	128(36.3)	6.91 (3.33)		33.03 (4.37)		18.98 (4.38)	
**Place of residence**			0.47		0.008		0.02
Rural	73(20.7)	7.10 (3.78)		33.16 (4.65)		20.66 (3.98)	
Township	61(17.3)	6.89 (3.62)		32.07 (4.80)		18.64 (4.49)	
Urban	219(62.0)	7.55 (3.29)		34.26 (4.06)		20.06 (3.82)	
**Educational level**			< 0.001		0.081		0.021
Junior high school or below	56(15.9)	5.68 (3.61)		32.46 (4.74)		19.07 (4.65)	
High school/technical secondary school	70(19.8)	7.17 (3.38)		32.84 (4.60)		18.93 (4.15)	
Associate degree/bachelor’s degree	191(54.1)	7.88 (3.31)		34.25 (4.27)		20.55 (3.68)	
Master’s degree or above	36(10.2)	7.42 (3.37)		33.92 (3.51)		20.03 (3.93)	
**Monthly income**			0.003		0.006		0.119
< 2000	73(20.7)	6.48 (3.40)		32.14 (4.74)		19.32 (4.10)	
2000–5000	104(29.5)	6.96 (3.56)		33.35 (4.36)		19.75 (4.27)	
5000–10,000	121(34.3)	7.73 (3.39)		34.42 (4.23)		19.98 (3.85)	
> 10,000	55(15.6)	8.36 (3.16)		34.56 (3.77)		21.05 (3.61)	
**Marital status**			0.012		0.013		0.001
Unmarried/other	104(29.5)	6.57 (3.72)		32.77 (4.96)		18.81 (3.80)	
Married	249(70.5)	7.67 (3.29)		34.02 (4.09)		20.41 (4.02)	
**Identity**			0.268		0.455		0.191
Patient	143(40.5)	7.66 (3.18)		33.84 (4.07)		19.58 (4.23)	
Accompanying family member	210(59.5)	7.13 (3.62)		33.53 (4.61)		20.19 (3.85)	
**Experience of MRI**			0.002		< 0.001		0.023
No	120(34.0)	6.56 (3.48)		32.32 (4.72)		19.19 (4.01)	
Yes	233(66.0)	7.75 (3.38)		34.34 (4.05)		20.33 (3.97)	
**Metallic implants in the body**			0.52		0.964		0.495
No	287(81.3)	7.40 (3.46)		33.63 (4.52)		19.99 (3.98)	
Yes	66(18.7)	7.09 (3.45)		33.74 (3.80)		19.71 (4.18)	
**Understand the material of the metallic implant in the body**			0.01		0.024		< 0.001
No	23(6.5)	5.26 (3.65)		31.91 (3.13)		16.57 (2.45)	
Yes	43(12.2)	8.07 (2.93)		34.72 (3.79)		21.40 (3.95)	
**Condition**			0.008		0.02		< 0.001
One of the above	69(19.5)	6.58 (3.09)		32.52 (4.92)		18.25 (4.11)	
None of the above	284(80.5)	7.53 (3.51)		33.93 (4.22)		20.35 (3.89)	
**History of drug allergies**			0.014		0.04		0.005
No	293(83.0)	7.50 (3.52)		33.84 (4.43)		20.19 (3.99)	
Yes	60(17.0)	6.57 (3.03)		32.75 (4.12)		18.70 (3.92)	

### Mean scores of knowledge, attitude, and practice by subgroup

The mean scores for knowledge, attitude, and practice among HCPs were 10.9 ± 2.00 (possible range: 0–28), 37.83 ± 4.07 (possible range: 12–60), and 22.34 ± 2.95 (possible range: 5–25), respectively. In contrast, among PFs, the mean scores for knowledge, attitude, and practice were 7.34 ± 3.45 (possible range: 0–28), 33.65 ± 4.39 (possible range: 12–60), and 19.94 ± 4.02 (possible range: 5–25), respectively.

### Knowledge, attitude, and practice dimensions

#### Knowledge dimension distribution

The analysis revealed significant knowledge gaps between HCPs and PFs. Among HCPs, 76% correctly identified that “MRI does not involve ionizing radiation” (**K1: False**), while 18.3% answered incorrectly. Additionally, 97.3% recognized that “prohibited electronic/metal items are not allowed in MRI rooms” (**K3: True**). However, 26.5% were uncertain about “burning sensations in tattooed areas during MRI” (**K14: Not sure**) (Supplementary Table S1). In contrast, PFs had notable misconceptions: 44.2% incorrectly believed “MRI uses ionizing radiation” (**K1: True**), and 53.3% misidentified the “MRI compatibility of coronary stents” (**K7**). Furthermore, 46.7% were unsure about “tattoo-related MRI risks” (**K14: Not sure**) (Supplementary Table S2).

### Attitude dimension distribution

HCPs showed confidence in MRI technology, with 94.7% agreeing that “MRI is highly accurate” (**A1**). However, 34.5% expressed safety concerns regarding “pediatric MRI examinations” (**A3: Disagree/Strongly disagree**), and 35% reported anxiety about “personal safety in MRI environments” (**A7: Agree/Strongly agree**) (Supplementary Table S3). PFs held more conservative views, with only 48.2% strongly agreeing that “MRI’s diagnostic accuracy” (**A1**) is high. Concerns about financial and safety issues were prominent, with 60.1% believing “MRI contrast agents may be harmful” (**A11**), and the same percentage considering “MRI costs unreasonable” (**A12**), exceeding HCPs by 28.2 and 25.8% points, respectively (Supplementary Table S4).

### Practice dimension distribution

HCPs generally followed safety protocols, with 72.2% indicating they “actively reported hazardous items pre-MRI” (**P2: Strongly agree/Agree**) and 60.2% stating they “read safety signage conscientiously” (**P1**). However, only 46.1% “routinely followed MRI safety updates” (**P4**) (Supplementary Table S5). PFs showed inconsistent compliance; while 79% reported “cooperating with pre-MRI screenings” (**P2: Strongly agree/Agree**), only 43.3% consistently “read safety warnings” (**P1**). Alarmingly, 36.3% were neutral about “daily MRI safety awareness” (**P4**), and 10.2% “rarely reminded others about MRI precautions” (**P5: Disagree/Strongly disagree**) (Supplementary Table S6).

### Univariate and multivariate analysis for Practice

For the Practice dimension, a total score of 19 points or below was defined as “low practice,” whereas a score of 20 points or above was classified as “adequate practice,”. According to this threshold, 78 (14.86%) HCPs and 163 (46.18%) PFs were classified as having low practice levels. A multivariate logistic regression analysis identified several significant predictors of effective practice: **(1) HCPs**: Significant predictors were identified as knowledge (OR = 1.189, 95% CI 1.045–1.354, *p* = 0.009), attitude (OR = 1.159, 95% CI 1.074–1.251, *p* < 0.001), work in internal medicine (OR = 0.364, *p* = 0.018), and involvement in other medical disciplines (OR = 0.451, *p* = 0.013) (Supplementary Table 7). **(2) PFs**: Key predictors included attitude (OR = 1.364, 95% CI 1.253–1.485, *p* < 0.001), residency in urban or township areas (OR = 0.291/0.387), educational attainment (associate/master’s degrees: OR = 5.148/4.193), and the absence of claustrophobia, renal dysfunction, or allergies (OR = 3.111, *p* = 0.007) (Supplementary Table 8).

### Spearman correlations between KAP

Significant interdomain correlations emerged in both subgroups, with distinct strength patterns (Tables 3 and 4). Among HCPs, knowledge showed moderate positive correlations with attitude (*r* = 0.354, *P* < 0.001) and weaker associations with practice (*r* = 0.230, *P* < 0.001). Attitude demonstrated the strongest linkage to practice (*r* = 0.444, *P* < 0.001), suggesting behavioral compliance was more influenced by perceptions than factual knowledge. In contrast, PFs exhibited stronger pairwise correlations: knowledge-attitude (*r* = 0.434 vs. HCP’s 0.354), knowledge-practice (*r* = 0.365 vs. 0.230), and notably attitude-practice (*r* = 0.632 vs. 0.444), all *P* < 0.001. This pattern indicates lay populations’ health behaviors are more holistically driven by both cognitive and affective factors compared to professionals.

**Table 3:** Spearman correlation analysis for healthcare providers (*n* = 525).

Spearman Correlation	Knowledge	Attitude	Practice
**Knowledge**	1.000		
**Attitude**	0.354 (*P* < 0.001)	1.000	
**Practice**	0.230 (*P* < 0.001)	0.444 (*P* < 0.001)	1.000

**Table 4:** Spearman correlation analysis for patients and their family (*n* = 353).

Spearman Correlation	Knowledge	Attitude	Practice
**Knowledge**	1.000		
**Attitude**	0.434 (*P* < 0.001)	1.000	
**Practice**	0.365 (*P* < 0.001)	0.632 (*P* < 0.001)	1.000

### Structural equation modeling analysis

**Fig. 1:**
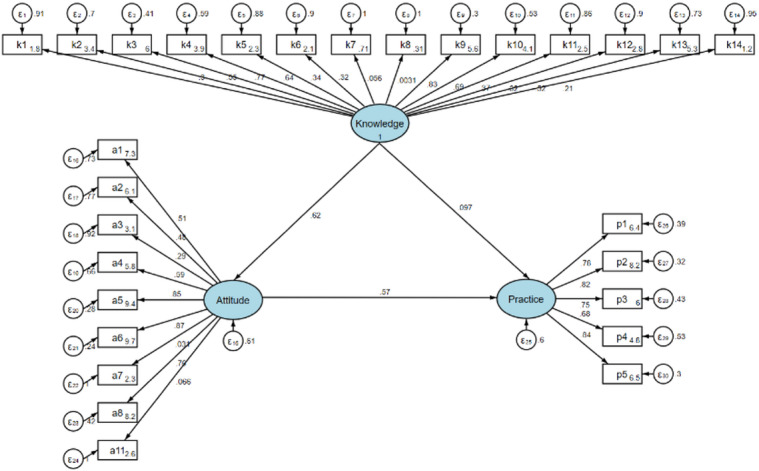
Structural equation model illustrating the hypothesized and estimated relationships among knowledge, attitude, and practice in healthcare providers (*n* = 525). Arrows represent the directional paths specified in the analytical model.

**Fig. 2:**
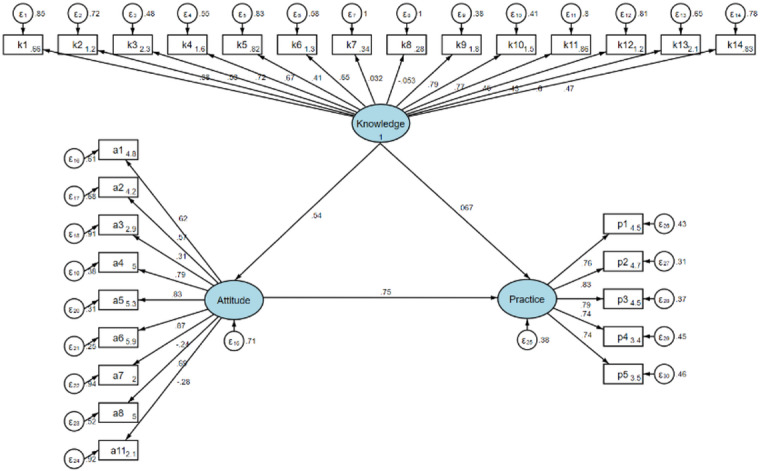
Structural equation model illustrating the hypothesized and estimated relationships among knowledge, attitude, and practice in patients and accompanying family members (*n* = 353). Arrows represent the directional paths specified in the analytical model.

Both subgroups demonstrated models with satisfactory fit. For HCPs, the fit indices indicated favorable outcomes (RMSEA = 0.051, SRMR = 0.042, TLI = 0.911, CFI = 0.923) (Supplementary Table 9). Among HCPs, knowledge exerted a strong direct effect on attitude (β = 0.622, 95% CI [0.558–0.687], *P* < 0.001); however, its direct effect on practice was not statistically significant. Attitude significantly influenced practice (β = 0.567, [0.464–0.670], *P* < 0.001), mediating 78.4% of the total effect of knowledge on practice (indirect effect: β = 0.353, [0.276–0.430], *P* < 0.001) (Table 5 & Fig. 1). In the case of PFs, the model exhibited excellent fit, as evidenced by the following indices: RMSEA = 0.049, SRMR = 0.038, TLI = 0.919, and CFI = 0.934 (Supplementary Table 10). Knowledge had a direct effect on attitude (β = 0.536, [0.448–0.623], *P* < 0.001), yet there was no direct effect observed on practice. Attitude demonstrated a more substantial direct effect on practice (β = 0.747, [0.662–0.832], *P* < 0.001), mediating 85.7% of the total effect of knowledge on practice (indirect effect: β = 0.400, [0.316–0.485], *P* < 0.001) (Table 6 & Fig. 2).

**Table 5:** SEM Results for Healthcare Providers (*n* = 525).

Modelpaths		Total Effects		Direct Effect		Indirect Effect	
		β (95%CI)	*P*	β (95%CI)	*P*	β (95%CI)	*P*
**Attitude**							
	**Knowledge**	0.622 (0.558, 0.687)	< 0.001	0.622 (0.558, 0.687)	< 0.001		
**Practice**							
	**Knowledge**	0.450 (0.370, 0.530)	< 0.001	0.097 (−0.014, 0.208)	0.087	0.353 (0.276, 0.430)	< 0.001
	**Attitude**	0.567 (0.464, 0.670)	< 0.001	0.567 (0.464, 0.670)	< 0.001		

**Table 6:** SEM Results for Patients and Their Family (*n* = 353).

Model Paths		Totaleffects		DirectEffect		Indirecteffect	
		β (95% CI)	*P*	β (95% CI)	*P*	β (95% CI)	*P*
**Attitude**							
	**Knowledge**	0.536 (0.448, 0.623)	< 0.001	0.536 (0.448, 0.623)	< 0.001		
**Practice**							
	**Knowledge**	0.467 (0.371, 0.562)	< 0.001	0.066 (−0.038, 0.172)	0.214	0.400 (0.316, 0.485)	< 0.001
	**Attitude**	0.747 (0.662, 0.832)	< 0.001	0.747 (0.662, 0.832)	< 0.001		

## Discussion

HCPs and PFs exhibited limited knowledge, moderate attitudes, and generally positive practices concerning MRI safety. SEM revealed that knowledge significantly influenced practice indirectly through attitudes, while the direct effect of knowledge on practice was not significant for either subgroup. These findings underscore the critical mediating role of attitudes in translating MRI safety knowledge into practical behaviors. Our regression models indicated that a one standard deviation increase in knowledge scores was associated with higher attitude scores, highlighting the indirect association between knowledge and MRI safety–related behavioral tendencies. This highlights the necessity for targeted educational interventions aimed at enhancing knowledge and fostering positive attitudes to ensure safe behaviors in MRI-related environments among healthcare professionals and the general public.

Previous research has demonstrated significant variability in MRI safety awareness among different populations, with healthcare professionals generally exhibiting a superior understanding^25^. This study elucidates the current levels of KAP among healthcare professionals, examinees, and accompanying family members, identifying areas of concern and implications for safe MRI practices. The findings reveal that while attitudes toward MRI safety are relatively positive, knowledge remains insufficient, particularly among non-professionals, which adversely affects the adoption of safe practices. The SEM analysis further highlights the indirect role of knowledge in shaping practices through attitudes, emphasizing the importance of both education and behavioral reinforcement.

A significant finding of this study is the inadequate knowledge of MRI safety among participants, especially non-professionals. Consistent with prior research, healthcare professionals exhibited higher levels of knowledge; however, gaps persisted in understanding contraindications and procedural safety^26,27^. Many participants, particularly PFs, expressed uncertainty regarding critical issues such as the risks associated with metallic implants, pacemakers, and the necessity for thorough screening prior to entering the MRI environment. Similar studies have indicated that the general public often lacks awareness of MRI-related risks, resulting in safety breaches^28,29^. Multivariate analysis demonstrated that knowledge was significantly associated with age and education, with younger and more educated individuals displaying a better understanding, while non-professionals exhibited considerable knowledge deficits. Given the established correlation between health literacy and safe medical behaviors, these findings suggest that current MRI safety education is inadequate for certain populations^8,29^.

Attitudes toward MRI safety were moderately positive; however, concerns regarding MRI procedures were prevalent, particularly among patients. Anxiety related to MRI, including fears about noise, scan duration, and potential risks from contrast agents, emerged as key factors influencing attitudes. These concerns align with previous research indicating that patient apprehension can hinder adherence to MRI safety guidelines^8,9^. Notably, the SEM analysis revealed that while knowledge strongly influenced attitudes, it did not exert a direct effect on practice for either healthcare professionals or patients. This suggests that fostering positive attitudes may be more critical for improving safety behaviors than merely providing information. This finding supports the notion that interventions should target psychological barriers to compliance, as attitude-driven motivation is essential for patient cooperation during MRI procedures^10,11^. Healthcare staff involved in MRI-related environments should be equipped with the skills to communicate MRI safety information effectively to examinees and accompanying family members, addressing common fears and misconceptions to enhance adherence to safety protocols.

Although practice scores were generally positive, a detailed analysis indicated that many participants did not actively seek additional MRI safety information or engage in precautionary measures beyond the basic requirements. This reflects a broader trend in healthcare studies where knowledge alone does not necessarily translate into proactive behaviors^30,31^. While most respondents adhered to basic MRI safety precautions, such as removing metal objects, fewer engaged in proactive information-seeking behaviors, indicating a passive approach to safety adherence. This raises concerns regarding the effectiveness of current educational strategies, as compliance with MRI safety recommendations necessitates both awareness and a willingness to actively engage with safety protocols. Demographic analysis revealed that individuals with metallic implants exhibited lower practice scores, reinforcing the need for targeted pre-examination counseling to prevent safety breaches.

The interrelationships among knowledge, attitudes, and practices underscore the necessity for an integrated approach to MRI safety education. Knowledge primarily influences practice through attitudes, emphasizing the importance of interventions that focus on both education and behavioral reinforcement. Subgroup analyses suggest that occupational background affects these relationships, with healthcare professionals demonstrating a more direct knowledge-attitude-practice link than patients and family members in MRI-related safety awareness and behavior patterns. From a systems perspective, these findings indicate deficiencies in public health education and patient engagement strategies. The persistence of safety misconceptions even among healthcare professionals underscores the need for ongoing professional training to ensure compliance with best practices. Other studies have demonstrated that standardized training programs can significantly enhance MRI safety adherence, further emphasizing the necessity for continuous education initiatives^32,33^.

To enhance MRI safety, a multi-faceted approach is essential. First, patient education programs should be expanded to address common knowledge gaps, with targeted initiatives for non-professionals, particularly those undergoing MRI scans or accompanying patients. These could include comprehensive pre-examination counseling, multimedia educational materials, and interactive learning modules tailored to various literacy levels. Second, healthcare professionals should receive training in effective risk communication to alleviate patient anxiety and improve adherence to safety measures^34^. This training should emphasize practical strategies for addressing patient concerns, reinforcing safety behaviors, and mitigating misconceptions. Third, institutional policies should incorporate structured safety monitoring systems, including mandatory pre-examination checklists and digital reminders to reinforce safe practices. Previous studies have suggested that structured safety monitoring systems may contribute to improved MRI safety management and incident prevention, highlighting the potential value of systematic institutional interventions^35^.

In the study institution, several routine MRI safety management measures are currently implemented to reduce potential risks in the MR environment. These include mandatory pre-examination safety screening questionnaires, on-site verbal safety confirmation by MRI technologists, visual safety signage in restricted areas, and standardized ferromagnetic object control procedures. In addition, healthcare providers and MRI-related staff are required to complete institutional MRI safety training programs prior to independent clinical practice, which cover basic MR safety principles, emergency procedures, and common risk scenarios. Despite these measures, the present findings indicate that gaps in knowledge and inconsistent safety-related behaviors still exist, particularly among non-radiology staff and non-professional participants. This suggests that routine training programs may benefit from periodic reinforcement, scenario-based simulation training, and standardized refresher courses to enhance long-term retention of safety knowledge. Furthermore, strengthening interdisciplinary communication between MRI technologists, referring clinicians, and nursing staff may help establish a more consistent safety culture across departments. These approaches may provide practical reference models for other hospitals aiming to improve MRI safety management systems and patient-centered safety education.

This study has several important limitations that should be acknowledged. First, the cross-sectional design limits the ability to establish causal or temporal relationships between knowledge, attitudes, and MRI safety–related practices. The observed associations reflect correlations at a single time point and should therefore be interpreted with caution. Second, although the questionnaire demonstrated acceptable reliability and construct validity, all data were collected using self-reported measures, which may be influenced by recall bias and social desirability bias. As a result, participants may have overestimated their knowledge levels or reported more favorable attitudes and practices than those occurring in real clinical settings. Third, the use of online convenience sampling and the single-center study design may have introduced selection bias, as individuals with higher digital literacy, stronger health awareness, or greater interest in MRI-related issues may have been more likely to participate. Consequently, the study sample may not fully represent broader patient, caregiver, or healthcare provider populations, particularly in rural or resource-limited settings. According to established MRI safety practice guidelines, healthcare institutions in different regions face varying challenges in implementing MRI safety protocols, including disparities in equipment maintenance, staff training resources, and safety monitoring systems. These structural differences may further limit the generalizability of the present findings to healthcare contexts with different organizational and resource conditions. Future studies may further incorporate standardized effect size measures to provide more quantitative comparisons of group differences^36^.

## Conclusion

HCPs and PFs exhibited insufficient knowledge, moderate attitudes, and relatively positive practices regarding MRI safety. The SEM analysis indicated that knowledge was indirectly associated with practice through attitude, highlighting the potential mediating role of attitudes in MRI safety–related behaviors. These findings suggest that educational and communication-based interventions may represent promising strategies for improving MRI safety awareness and behavioral compliance. However, further longitudinal and interventional studies are needed to verify causal relationships and to evaluate the effectiveness of targeted educational programs in improving real-world MRI safety outcomes.

## Supplementary Information

Below is the link to the electronic supplementary material.

Supplementary Material 1

Supplementary Material 2

## Data Availability

All data generated or analysed during this study are included in this published article.
