# Supplementary material for: Knowledge attitudes and practices regarding MRI safety among healthcare providers and patients/family members in China
Source: Sci Rep. 2026 Mar 23;16:14571. doi: 10.1038/s41598-026-44648-5 (PMC13153162; doi:10.1038/s41598-026-44648-5)
Supplement: Supplementary file 1 — Supplementary Material 1 [file 41598_2026_44648_MOESM1_ESM.docx]

**Knowledge, Attitudes, and Practices Regarding MRI Safety Among Healthcare Providers, Patients and Families**

| **Part 1 Basic Information** | | |
| --- | --- | --- |
| **1. Your gender:** | a. Male | b. Female |
| **2. Your age: ____** years old. (Numbers only; please set numerical validation to integers in the questionnaire system.) | | |
| **3. Your place of residence:** | a. Rural b. Township c. Urban | |
| **4. Your educational level:** | a. Junior high school or below b. High school/Technical secondary school c. Bachelor's degree/Associate degree d. Master's degree or above | |
| **5.Your monthly income (in RMB):** | a. <2000  b.2000-5000  c.5000-10000  d.10000-20000  e.>20000 | |
| **6.Your marital status:** | a. Unmarried b. Married c. Divorced d. Other | |
| **7.Your identity:** | a. Healthcare professional or medical student b. Patient (If selected, skip to Question 12.) c. Accompanying family member (If selected, skip to Question 12.) | |
| **8.Your department:** | a. Radiology/Imaging Department b. Internal Medicine c. Surgery d. Other departments | |
| **9.Your professional title level:** | a. None (including medical students, interns, etc.) b. Junior (including resident doctors, nurses, junior technicians, etc.) c. Intermediate (including attending physicians, senior nurses, senior technicians, etc.) d. Associate senior (including associate chief physicians, associate chief nurses, associate chief technicians, etc.) e. Senior (including chief physicians, chief nurses, chief technicians, etc.) | |
| **10.Years of work experience in a medical institution:** | a. <1 year b. 1-5 years c. 6-10 years d. >10 years | |
| **11.Level of your hospital:** | a. Provincial/ministerial level b. Municipal level c. County level d. Township level or below | |
| **12.Have you ever undergone a magnetic resonance imaging (MRI) examination?** | a. Yes b. No | |
| **13.Do you have any metallic implants in your body (including pacemakers, cochlear implants or hearing aids, continuous glucose monitors, insulin pumps, pain pumps, aneurysm clips, stents, metallic valves, internal fixation devices, intrauterine devices, orthodontic braces, etc.)?** | a. Yes b. No (If selected, skip to Question 15.) | |
| **14.Do you know the material of the metallic implant in your body (e.g., stainless steel, alloy, titanium, etc.)?** | a. Yes b. No | |
| **15.Do you have any of the following conditions?** | a. Claustrophobia b. Impaired renal function c. Allergy-prone constitution d. None of the above | |
| **16.Do you have a history of drug or contrast agent allergies?** | a. Yes b. No | |

| **Part 2 Knowledge of Magnetic Resonance Imaging (MRI)** | | | |
| --- | --- | --- | --- |
| **Please indicate your level of understanding regarding the following statements:** | | | |
| **1.MRI (Magnetic Resonance Imaging) involves ionizing radiation.** | a. True | b. False | c. Not sure |
| 1. **MRI provides better imaging of structures such as the brain, abdominal and pelvic organs (liver, gallbladder, spleen, pancreas, kidneys, uterus, prostate, etc.), joints, and muscles compared to CT.** | a. True | b. False | c. Not sure |
| **3.Electronic devices such as mobile phones and watches, as well as metal jewelry such as necklaces, earrings, and rings, are not allowed in the MRI examination room.** | a. True | b. False | c. Not sure |
| **4.Wheelchairs, stretchers, oxygen cylinders, and ECG monitors cannot be used inside the MRI examination room.** | a. True | b. False | c. Not sure |
| **5.The carts and emergency supplies used by medical emergency personnel can directly enter the MRI examination room.** | a. True | b. False | c. Not sure |
| **6.Patients with implanted cardiac pacemakers cannot undergo an MRI examination.** | a. True | b. False | c. Not sure |
| **7.Patients with implanted coronary or peripheral vascular stents cannot undergo an MRI examination.** | a. True | b. False | c. Not sure |
| **8.A safety check is required upon entering the MRI reception area.** | a. True | b. False | c. Not sure |
| 1. **A screening for contraindicated items must be conducted before entering the MRI examination room.** | a. True | b. False | c. Not sure |
| **10.All individuals accompanying patients into the MRI examination room must undergo the same screening for contraindicated items.** | a. True | b. False | c. Not sure |
| **11.If the MRI machine has not been turned on, there is no need to screen for contraindicated items before entering the examination room.** | a. True | b. False | c. Not sure |
| **12.Medical staff can freely enter and exit the MRI examination room during the examination.** | a. True | b. False | c. Not sure |
| **13.The body must remain still during an MRI examination.** | a. True | b. False | c. Not sure |
| **14.Patients with large tattoos, dyed hair, or makeup may experience a burning sensation in the affected areas during an MRI examination.** | a. True | b. False | c. Not sure |

| **Part 3 Attitudes Toward Magnetic Resonance Imaging (MRI)** | | | | | |
| --- | --- | --- | --- | --- | --- |
| **1.I believe MRI is highly accurate, and I am willing to undergo the examination.** | a. Strongly agree | b. Agree | c. Neutral | d. Disagree | e. Strongly disagree |
| **2.I believe MRI is a well-established technology that does not pose a health risk.** | a. Strongly agree | b. Agree | c. Neutral | d. Disagree | e. Strongly disagree |
| **3.I believe there are no safety concerns when children undergo MRI examinations.** | a. Strongly agree | b. Agree | c. Neutral | d. Disagree | e. Strongly disagree |
| **4.I believe signing an informed consent form before undergoing an MRI examination is necessary.** | a. Strongly agree | b. Agree | c. Neutral | d. Disagree | e. Strongly disagree |
| **5.I believe screening for contraindicated items before an MRI examination is essential.** | a. Strongly agree | b. Agree | c. Neutral | d. Disagree | e. Strongly disagree |
| **6.I believe safety reminders in the MRI examination area are necessary.** | a. Strongly agree | b. Agree | c. Neutral | d. Disagree | e. Strongly disagree |
| **7.I feel concerned about my safety when entering an MRI-related area.** | a. Strongly agree | b. Agree | c. Neutral | d. Disagree | e. Strongly disagree |
| **8.I believe it is important to understand MRI safety requirements.** | a. Strongly agree | b. Agree | c. Neutral | d. Disagree | e. Strongly disagree |
| **9.I think MRI examinations take too long.** | a. Strongly agree | b. Agree | c. Neutral | d. Disagree | e. Strongly disagree |
| **10.I feel concerned about the noise generated during an MRI examination.** | a. Strongly agree | b. Agree | c. Neutral | d. Disagree | e. Strongly disagree |
| **11.I worry that the contrast agent used in special MRI examinations may be harmful to my body.** | a. Strongly agree | b. Agree | c. Neutral | d. Disagree | e. Strongly disagree |
| **12.I believe MRI examinations are too expensive and unreasonable.** | a. Strongly agree | b. Agree | c. Neutral | d. Disagree | e. Strongly disagree |

c

| **Part 4 MRI Safety Practices** | | | | | |
| --- | --- | --- | --- | --- | --- |
| **1.Before entering an MRI-related area, I carefully read the safety warning signs on the walls.** | a. Strongly agree | b. Agree | c. Neutral | d. Disagree | e. Strongly disagree |
| **2.Before entering the MRI examination room, I actively cooperate with staff and inform them if I am carrying any potentially hazardous items.** | a. Strongly agree | b. Agree | c. Neutral | d. Disagree | e. Strongly disagree |
| **3.I proactively ask medical staff about any concerns I have regarding the MRI examination.** | a. Strongly agree | b. Agree | c. Neutral | d. Disagree | e. Strongly disagree |
| **4.In my daily life, I pay close attention to common knowledge about MRI safety.** | a. Strongly agree | b. Agree | c. Neutral | d. Disagree | e. Strongly disagree |
| **5.When I learn that someone around me needs to undergo an MRI examination, I actively remind them to pay attention to relevant safety information.** | a. Strongly agree | b. Agree | c. Neutral | d. Disagree | e. Strongly disagree |

| **Thank you again for filling out our questionnaire, the information you provided will be valuable to us in the future!**  **Thank you for filling out our questionnaire！**  If you have any comments and suggestions on this survey, we would be honored to hear your voice.  Opinions and Suggestions： （optional）  In order to this questionnaire research can actually play a role in promoting the smooth development of the future return visit, if you are willing to leave your contact information, we would be grateful!  Your phone number： （optional） |
| --- |
