# Supplementary material for: Knowledge attitudes and practices regarding MRI safety among healthcare providers and patients/family members in China
Source: Sci Rep. 2026 Mar 23;16:14571. doi: 10.1038/s41598-026-44648-5 (PMC13153162; doi:10.1038/s41598-026-44648-5)
Supplement: Supplementary file 2 — Supplementary Material 2 [file 41598_2026_44648_MOESM2_ESM.docx]

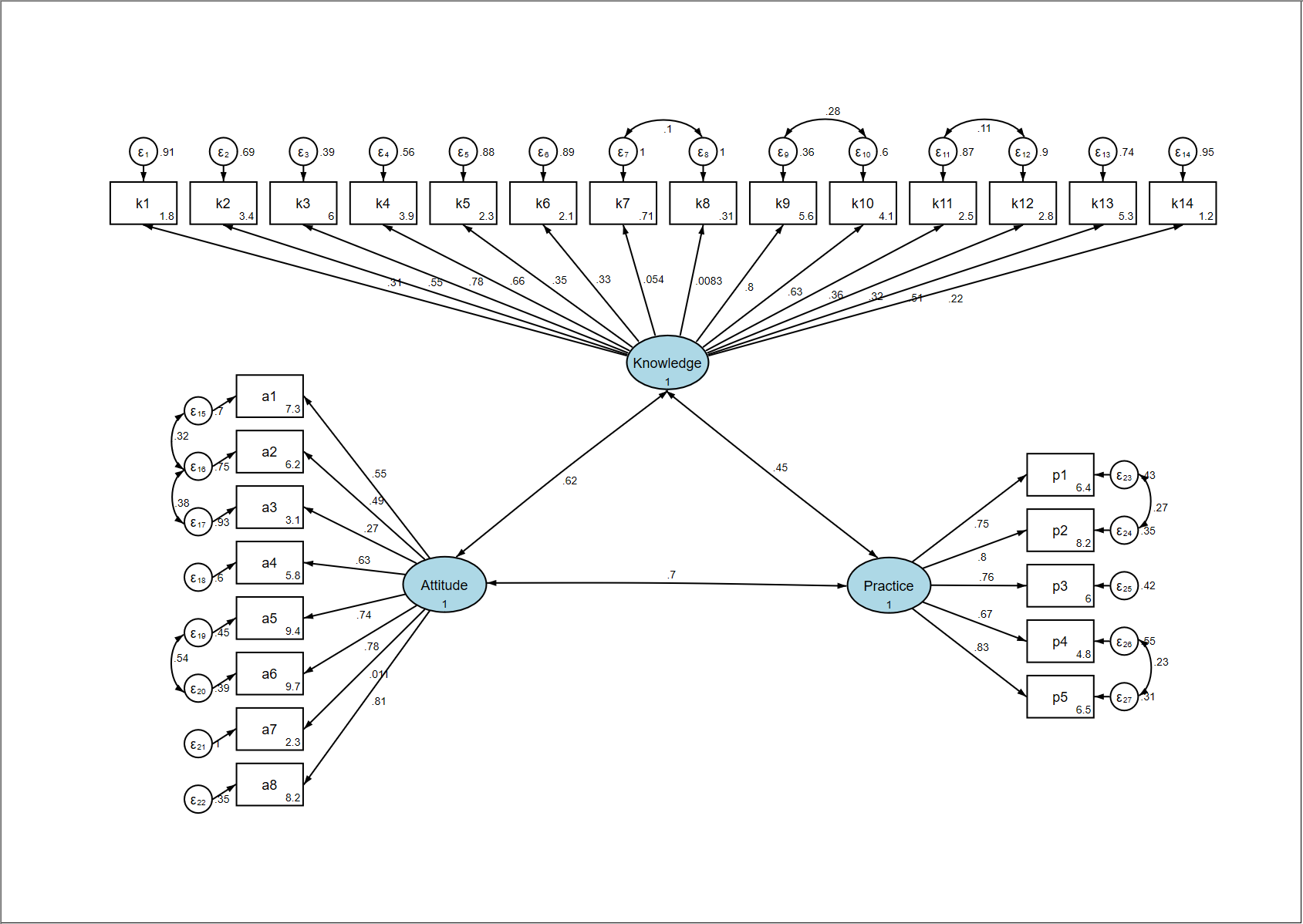


**Supplementary Figure 1. Measurement model (confirmatory factor analysis) for healthcare providers and medical students (N = 525).**


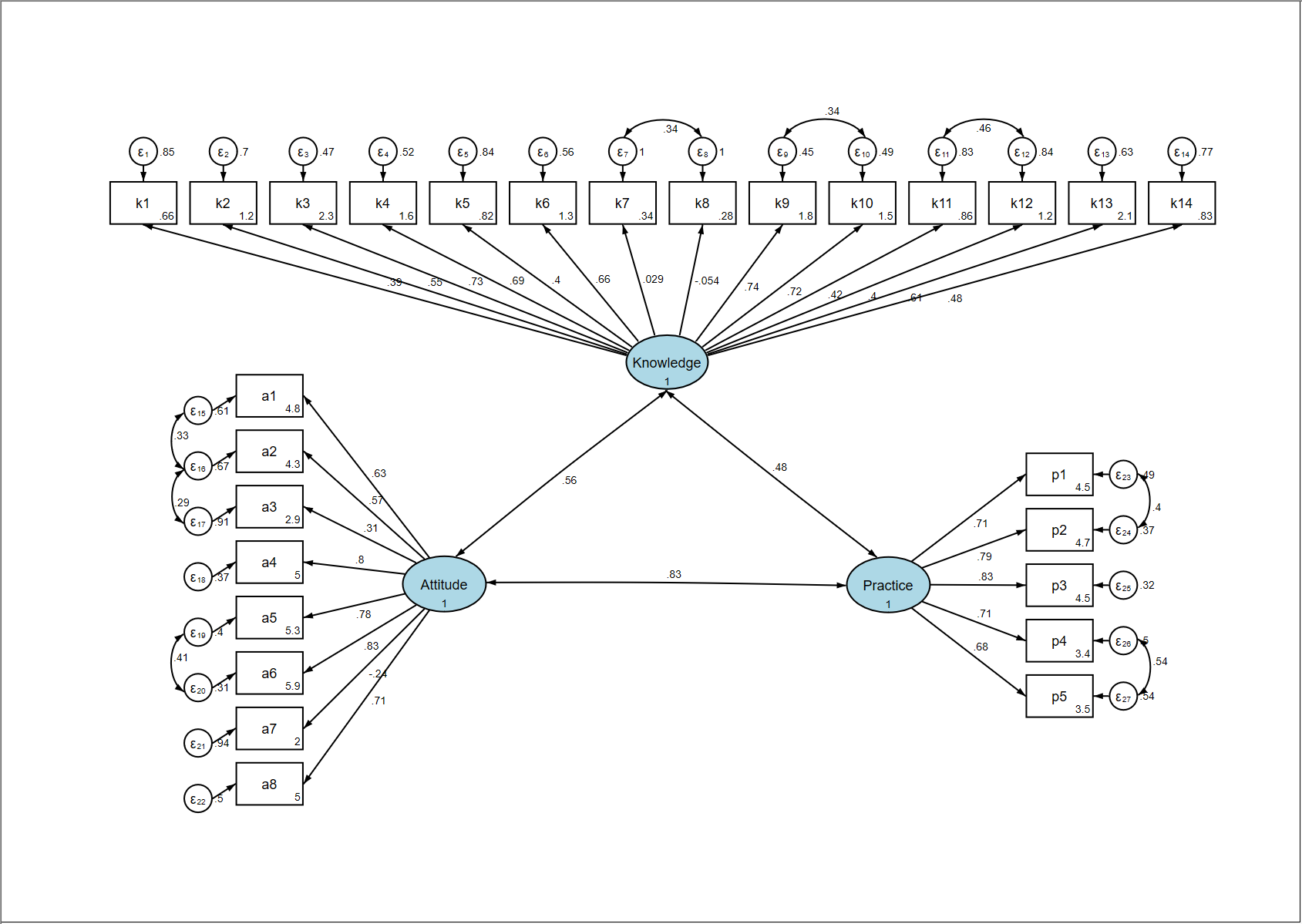


**Supplementary Figure 2. Measurement model (confirmatory factor analysis) for patients and accompanying family members (N = 353).**

**Supplementary Table 1. Distribution of Knowledge Dimension Responses for Healthcare Providers (N=525).**

| **Knowledge Dimension** | **True** | **False** | **Not sure** |
| --- | --- | --- | --- |
| **1. MRI (Magnetic Resonance Imaging) involves ionizing radiation.** | 96(18.3%) | 399(76%) | 30(5.7%) |
| **2. MRI provides better imaging of structures such as the brain, abdominal and pelvic organs (liver, gallbladder, spleen, pancreas, kidneys, uterus, prostate, etc.), joints, and muscles compared to CT.** | 484(92.2%) | 14(2.7%) | 27(5.1%) |
| **3. Electronic devices such as mobile phones and watches, as well as metal jewelry such as necklaces, earrings, and rings, are not allowed in the MRI examination room.** | 511(97.3%) | 4(0.8%) | 10(1.9%) |
| **4. Wheelchairs, stretchers, oxygen cylinders, and ECG monitors cannot be used inside the MRI examination room.** | 492(93.7%) | 13(2.5%) | 20(3.8%) |
| **5. The carts and emergency supplies used by medical emergency personnel can directly enter the MRI examination room.** | 51(9.7%) | 444(84.6%) | 30(5.7%) |
| **6. Patients with implanted cardiac pacemakers cannot undergo an MRI examination.** | 426(81.1%) | 62(11.8%) | 37(7%) |
| **7. Patients with implanted coronary or peripheral vascular stents cannot undergo an MRI examination.** | 304(57.9%) | 175(33.3%) | 46(8.8%) |
| **8. A safety check is required upon entering the MRI reception area.** | 453(86.3%) | 45(8.6%) | 27(5.1%) |
| **A screening for contraindicated items must be conducted before entering the MRI examination room.** | 509(97%) | 5(1%) | 11(2.1%) |
| **10. All individuals accompanying patients into the MRI examination room must undergo the same screening for contraindicated items.** | 496(94.5%) | 11(2.1%) | 18(3.4%) |
| **11. If the MRI machine has not been turned on, there is no need to screen for contraindicated items before entering the examination room.** | 49(9.3%) | 454(86.5%) | 22(4.2%) |
| **12. Medical staff can freely enter and exit the MRI examination room during the examination.** | 41(7.8%) | 467(89%) | 17(3.2%) |
| **13. The body must remain still during an MRI examination.** | 507(96.6%) | 5(1%) | 13(2.5%) |
| **14. Patients with large tattoos, dyed hair, or makeup may experience a burning sensation in the affected areas during an MRI examination.** | 313(59.6%) | 73(13.9%) | 139(26.5%) |

**Supplementary Table 2. Distribution of Knowledge Dimension Responses for Patients and Their Family (N=353).**

| **Knowledge Dimension** | **True** | **False** | **Not sure** |
| --- | --- | --- | --- |
| **1. MRI (Magnetic Resonance Imaging) involves ionizing radiation.** | 156(44.2%) | 107(30.3%) | 90(25.5%) |
| **2. MRI provides better imaging of structures such as the brain, abdominal and pelvic organs (liver, gallbladder, spleen, pancreas, kidneys, uterus, prostate, etc.), joints, and muscles compared to CT.** | 215(60.9%) | 24(6.8%) | 114(32.3%) |
| **3. Electronic devices such as mobile phones and watches, as well as metal jewelry such as necklaces, earrings, and rings, are not allowed in the MRI examination room.** | 298(84.4%) | 14(4%) | 41(11.6%) |
| **4. Wheelchairs, stretchers, oxygen cylinders, and ECG monitors cannot be used inside the MRI examination room.** | 250(70.8%) | 17(4.8%) | 86(24.4%) |
| **5. The carts and emergency supplies used by medical emergency personnel can directly enter the MRI examination room.** | 93(26.3%) | 141(39.9%) | 119(33.7%) |
| **6. Patients with implanted cardiac pacemakers cannot undergo an MRI examination.** | 216(61.2%) | 24(6.8%) | 113(32%) |
| **7. Patients with implanted coronary or peripheral vascular stents cannot undergo an MRI examination.** | 188(53.3%) | 36(10.2%) | 129(36.5%) |
| **8. A safety check is required upon entering the MRI reception area.** | 246(69.7%) | 26(7.4%) | 81(22.9%) |
| **9. A screening for contraindicated items must be conducted before entering the MRI examination room.** | 269(76.2%) | 17(4.8%) | 67(19%) |
| **10. All individuals accompanying patients into the MRI examination room must undergo the same screening for contraindicated items.** | 249(70.5%) | 28(7.9%) | 76(21.5%) |
| **11. If the MRI machine has not been turned on, there is no need to screen for contraindicated items before entering the examination room.** | 105(29.7%) | 150(42.5%) | 98(27.8%) |
| **12. Medical staff can freely enter and exit the MRI examination room during the examination.** | 70(19.8%) | 204(57.8%) | 79(22.4%) |
| **13. The body must remain still during an MRI examination.** | 287(81.3%) | 11(3.1%) | 55(15.6%) |
| **14. Patients with large tattoos, dyed hair, or makeup may experience a burning sensation in the affected areas during an MRI examination.** | 144(40.8%) | 44(12.5%) | 165(46.7%) |

**Supplementary Table 3. Distribution of Attitude Dimension Responses for Healthcare Providers (N=525).**

| **Attitude Dimension** | **Strongly agree** | **Agree** | **Neutral** | **Disagree** | **Strongly disagree** |
| --- | --- | --- | --- | --- | --- |
| **1. I believe MRI is highly accurate, and I am willing to undergo the examination.** | 324(61.7%) | 173(33%) | 25(4.8%) | 2(0.4%) | 1(0.2%) |
| **2. I believe MRI is a well-established technology that does not pose a health risk.** | 286(54.5%) | 193(36.8%) | 35(6.7%) | 9(1.7%) | 2(0.4%) |
| **3. I believe there are no safety concerns when children undergo MRI examinations.** | 179(34.1%) | 141(26.9%) | 116(22.1%) | 63(12%) | 26(5%) |
| **4. I believe signing an informed consent form before undergoing an MRI examination is necessary.** | 313(59.6%) | 162(30.9%) | 39(7.4%) | 5(1%) | 6(1.1%) |
| **5. I believe screening for contraindicated items before an MRI examination is essential.** | 452(86.1%) | 59(11.2%) | 10(1.9%) | 1(0.2%) | 3(0.6%) |
| **6. I believe safety reminders in the MRI examination area are necessary.** | 450(85.7%) | 64(12.2%) | 6(1.1%) | 3(0.6%) | 2(0.4%) |
| **7. I feel concerned about my safety when entering an MRI-related area.** | 92(17.5%) | 92(17.5%) | 153(29.1%) | 125(23.8%) | 63(12%) |
| **8. I believe it is important to understand MRI safety requirements.** | 400(76.2%) | 108(20.6%) | 13(2.5%) | 1(0.2%) | 3(0.6%) |
| **9. I think MRI examinations take too long.** | 110(21%) | 237(45.1%) | 150(28.6%) | 17(3.2%) | 11(2.1%) |
| **10. I feel concerned about the noise generated during an MRI examination.** | 76(14.5%) | 145(27.6%) | 224(42.7%) | 58(11%) | 22(4.2%) |
| **11. I worry that the contrast agent used in special MRI examinations may be harmful to my body.** | 61(11.6%) | 152(29%) | 182(34.7%) | 99(18.9%) | 31(5.9%) |
| **12. I believe MRI examinations are too expensive and unreasonable.** | 70(13.3%) | 110(21%) | 226(43%) | 91(17.3%) | 28(5.3%) |

**Supplementary Table 4. Distribution of Attitude Dimension Responses for Patients and Their Family (N=353).**

| **Attitude Dimension** | **Strongly agree** | **Agree** | **Neutral** | **Disagree** | **Strongly disagree** |
| --- | --- | --- | --- | --- | --- |
| **1. I believe MRI is highly accurate, and I am willing to undergo the examination.** | 170(48.2%) | 124(35.1%) | 45(12.7%) | 8(2.3%) | 6(1.7%) |
| **2. I believe MRI is a well-established technology that does not pose a health risk.** | 140(39.7%) | 122(34.6%) | 70(19.8%) | 15(4.2%) | 6(1.7%) |
| **3. I believe there are no safety concerns when children undergo MRI examinations.** | 86(24.4%) | 83(23.5%) | 103(29.2%) | 62(17.6%) | 19(5.4%) |
| **4. I believe signing an informed consent form before undergoing an MRI examination is necessary.** | 156(44.2%) | 135(38.2%) | 52(14.7%) | 5(1.4%) | 5(1.4%) |
| **5. I believe screening for contraindicated items before an MRI examination is essential.** | 187(53%) | 126(35.7%) | 31(8.8%) | 2(0.6%) | 7(2%) |
| **6. I believe safety reminders in the MRI examination area are necessary.** | 197(55.8%) | 124(35.1%) | 25(7.1%) | 3(0.8%) | 4(1.1%) |
| **7. I feel concerned about my safety when entering an MRI-related area.** | 103(29.2%) | 104(29.5%) | 99(28%) | 29(8.2%) | 18(5.1%) |
| **8. I believe it is important to understand MRI safety requirements.** | 167(47.3%) | 135(38.2%) | 38(10.8%) | 7(2%) | 6(1.7%) |
| **9. I think MRI examinations take too long.** | 98(27.8%) | 120(34%) | 110(31.2%) | 16(4.5%) | 9(2.5%) |
| **10. I feel concerned about the noise generated during an MRI examination.** | 90(25.5%) | 123(34.8%) | 107(30.3%) | 21(5.9%) | 12(3.4%) |
| **11. I worry that the contrast agent used in special MRI examinations may be harmful to my body.** | 94(26.6%) | 125(35.4%) | 89(25.2%) | 30(8.5%) | 15(4.2%) |
| **12. I believe MRI examinations are too expensive and unreasonable.** | 91(25.8%) | 121(34.3%) | 101(28.6%) | 25(7.1%) | 15(4.2%) |

**Supplementary Table 5. Distribution of Practice** **Dimension Responses for Healthcare Providers (N=525).**

| **Practice Dimension** | **Strongly agree** | **Agree** | **Neutral** | **Disagree** | **Strongly disagree** |
| --- | --- | --- | --- | --- | --- |
| **1. Before entering an MRI-related area, I carefully read the safety warning signs on the walls.** | 316(60.2%) | 160(30.5%) | 43(8.2%) | 5(1%) | 1(0.2%) |
| **2. Before entering the MRI examination room, I actively cooperate with staff and inform them if I am carrying any potentially hazardous items.** | 379(72.2%) | 124(23.6%) | 21(4%) | 0 (0%) | 1(0.2%) |
| **3. I proactively ask medical staff about any concerns I have regarding the MRI examination.** | 313(59.6%) | 153(29.1%) | 50(9.5%) | 8(1.5%) | 1(0.2%) |
| **4. In my daily life, I pay close attention to common knowledge about MRI safety.** | 242(46.1%) | 162(30.9%) | 106(20.2%) | 13(2.5%) | 2(0.4%) |
| **5. When I learn that someone around me needs to undergo an MRI examination, I actively remind them to pay attention to relevant safety information.** | 318(60.6%) | 162(30.9%) | 39(7.4%) | 5(1%) | 1(0.2%) |

**Supplementary Table 6. Distribution of Practice Dimension Responses for Patients and Their Family (N=353).**

| **Practice Dimension** | **Strongly agree** | **Agree** | **Neutral** | **Disagree** | **Strongly disagree** |
| --- | --- | --- | --- | --- | --- |
| **1. Before entering an MRI-related area, I carefully read the safety warning signs on the walls.** | 153(43.3%) | 119(33.7%) | 67(19%) | 8(2.3%) | 6(1.7%) |
| **2. Before entering the MRI examination room, I actively cooperate with staff and inform them if I am carrying any potentially hazardous items.** | 162(45.9%) | 122(34.6%) | 54(15.3%) | 11(3.1%) | 4(1.1%) |
| **3. I proactively ask medical staff about any concerns I have regarding the MRI examination.** | 139(39.4%) | 122(34.6%) | 81(22.9%) | 6(1.7%) | 5(1.4%) |
| **4. In my daily life, I pay close attention to common knowledge about MRI safety.** | 105(29.7%) | 78(22.1%) | 128(36.3%) | 33(9.3%) | 9(2.5%) |
| **5. When I learn that someone around me needs to undergo an MRI examination, I actively remind them to pay attention to relevant safety information.** | 124(35.1%) | 93(26.3%) | 93(26.3%) | 36(10.2%) | 7(2%) |

**Supplementary Table 7. Univariate and Multivariate Logistic regression analysis for Practice Dimension of Healthcare Providers (N=525).**

| **Practice** | Univariate analysis | P | Multivariate analysis | P |
| --- | --- | --- | --- | --- |
|  | OR (95%CI) |  | OR (95%CI) |  |
| **Knowledge** | 1.400 (1.248,1.571) | <0.001 | 1.189 (1.045,1.354) | 0.009 |
| **Attitude** | 1.235 (1.155,1.320) | <0.001 | 1.159 (1.074,1.251) | <0.001 |
| **Gender** |  |  |  |  |
| Male |  |  |  |  |
| Female | 1.260 (0.766,2.052) | 0.356 |  |  |
| **Age** |  |  |  |  |
| 25 or below |  |  |  |  |
| 26~45 | 1.213 (0.734,2.042) | 0.457 |  |  |
| More than 45 | 1.460 (0.484,6.326) | 0.550 |  |  |
| **Place of residence** |  |  |  |  |
| Rural |  |  |  |  |
| Township | 0.834 (0.402,1.780) | 0.631 |  |  |
| Urban | 1.270 (0.715,2.205) | 0.403 |  |  |
| **Educational level** |  |  |  |  |
| Associate degree /bachelor’s degree |  |  |  |  |
| Master’s degree or above | 0.655 (0.368,1.218) | 0.164 |  |  |
| **Monthly income** |  |  |  |  |
| <2000 |  |  |  |  |
| 2000-5000 | 1.558 (0.756,3.547) | 0.256 |  |  |
| 5000-10000 | 1.145 (0.634,2.150) | 0.662 |  |  |
| >10000 | 1.193 (0.553,2.875) | 0.672 |  |  |
| **Marital status** |  |  |  |  |
| Unmarried/other |  |  |  |  |
| Married | 1.092 (0.659,1.851) | 0.736 |  |  |
| **Department** |  |  |  |  |
| Radiology/Imaging Department |  |  |  |  |
| Internal medicine | 0.248 (0.116,0.533) | <0.001 | 0.364 (0.158,0.838) | 0.018 |
| Surgery | 1.073 (0.287,6.992) | 0.927 | 1.292 (0.276,6.039) | 0.745 |
| Other department | 0.303 (0.165,0.537) | <0.001 | 0.451 (0.240,0.846) | 0.013 |
| **Professional title level** |  |  |  |  |
| None |  |  |  |  |
| Junior | 1.754 (0.836,4.151) | 0.164 |  |  |
| Intermediate | 1.225 (0.671,2.355) | 0.523 |  |  |
| Associate senior or above | 1.186 (0.474,3.609) | 0.737 |  |  |
| **Years of work experience** |  |  |  |  |
| ＜1 year |  |  |  |  |
| 1-5 years | 1.180 (0.569,2.701) | 0.673 |  |  |
| 6-10 years | 1.230 (0.574,2.949) | 0.616 |  |  |
| >10 years | 1.498 (0.798,2.993) | 0.227 |  |  |
| **Level of the hospital** |  |  |  |  |
| Provincial/ministerial level |  |  |  |  |
| Municipal level | 0.887 (0.509,1.588) | 0.679 |  |  |
| County level | 1.070 (0.528,2.357) | 0.858 |  |  |
| Township level or below | 1.265 (0.416,5.504) | 0.712 |  |  |
| **Experience of MRI** |  |  |  |  |
| No |  |  |  |  |
| Yes | 1.843 (1.127,3.065) | 0.016 | 1.455 (0.839,2.521) | 0.182 |
| **Metallic implants in the body** |  |  |  |  |
| No |  |  |  |  |
| Yes | 0.821 (0.299,2.893) | 0.727 |  |  |
| **Condition** |  |  |  |  |
| One of the above |  |  |  |  |
| None of the above | 1.266 (0.529,2.704) | 0.565 |  |  |
| **History of drug allergies** |  |  |  |  |
| No |  |  |  |  |
| Yes | 0.500 (0.214,1.308) | 0.128 |  |  |

**Supplementary Table 8. Univariate and Multivariate Logistic regression analysis for Practice Dimension of Patients and Their Family (N=353).**

| **Practice Dimension** | Univariate analysis | P | Multivariate analysis | P |
| --- | --- | --- | --- | --- |
|  | OR (95%CI) |  | OR (95%CI) |  |
| **Knowledge** | 1.235 (1.152,1.324) | <0.001 | 1.075 (0.986,1.173) | 0.101 |
| **Attitude** | 1.366 (1.269,1.469) | <0.001 | 1.364 (1.253,1.485) | <0.001 |
| **Gender** |  |  |  |  |
| Male |  |  |  |  |
| Female | 0.761 (0.500,1.158) | 0.203 |  |  |
| **Age** |  |  |  |  |
| 25 or below |  |  |  |  |
| 26~45 | 2.091 (1.140,3.868) | 0.018 | 2.182 (0.700,6.805) | 0.179 |
| More than 45 | 0.957 (0.511,1.799) | 0.891 | 1.481 (0.452,4.859) | 0.517 |
| **Place of residence** |  |  |  |  |
| Rural |  |  |  |  |
| Township | 0.490 (0.243,0.972) | 0.043 | 0.387 (0.151,0.996) | 0.049 |
| Urban | 0.799 (0.463,1.364) | 0.414 | 0.291 (0.119,0.713) | 0.007 |
| **Educational level** |  |  |  |  |
| Junior high school or below |  |  |  |  |
| High school /technical secondary school | 1.301 (0.639,2.673) | 0.469 | 2.786 (0.951,8.166) | 0.062 |
| Associate degree /bachelor’s degree | 2.339 (1.279,4.350) | 0.006 | 5.148 (1.666,15.905) | 0.004 |
| Master’s degree or above | 2.164 (0.930,5.152) | 0.076 | 4.193 (1.064,16.530) | 0.041 |
| **Monthly income** |  |  |  |  |
| <2000 |  |  |  |  |
| 2000-5000 | 1.219 (0.670,2.225) | 0.518 | 0.640 (0.278,1.476) | 0.296 |
| 5000-10000 | 1.179 (0.659,2.115) | 0.578 | 0.334 (0.129,0.867) | 0.024 |
| >10000 | 2.232 (1.089,4.682) | 0.030 | 0.643 (0.213,1.935) | 0.432 |
| **Marital status** |  |  |  |  |
| Unmarried/other |  |  |  |  |
| Married | 1.636 (1.034,2.601) | 0.036 | 1.343 (0.586,3.078) | 0.486 |
| **Identity** |  |  |  |  |
| Patient |  |  |  |  |
| Accompanying family member | 1.326 (0.866,2.034) | 0.195 |  |  |
| **Experience of MRI** |  |  |  |  |
| No |  |  |  |  |
| Yes | 1.262 (0.812,1.965) | 0.301 |  |  |
| **Metallic implants in the body** |  |  |  |  |
| No |  |  |  |  |
| Yes | 0.768 (0.448,1.314) | 0.335 |  |  |
| **Condition** |  |  |  |  |
| One of the above |  |  |  |  |
| None of the above | 3.094 (1.789,5.494) | <0.001 | 3.111 (1.363,7.100) | 0.007 |
| **History of drug allergies** |  |  |  |  |
| No |  |  |  |  |
| Yes | 0.510 (0.287,0.893) | 0.020 | 1.415 (0.619,3.233) | 0.411 |

**Supplementary Table 9. SEM fit indicators for Healthcare Providers (N=525).**

| **Indicators** | **Reference** | **Results** |
| --- | --- | --- |
| RMSEA | <0.08 | 0.073 |
| SRMR | <0.08 | 0.068 |
| TLI | >0.80 | 0.800 |
| CFI | >0.80 | 0.816 |

**Supplementary Table 10. SEM fit indicators for Patients and Their Family (N=353).**

| **Indicators** | **Reference** | **Results** |
| --- | --- | --- |
| RMSEA | <0.08 | 0.088 |
| SRMR | <0.08 | 0.075 |
| TLI | >0.80 | 0.762 |
| CFI | >0.80 | 0.782 |
